# Association between circulating exhausted CD4^+^ T cells with poor meningococcal C conjugate vaccine antibody response in HIV-infected children and adolescents

**DOI:** 10.6061/clinics/2021/e2902

**Published:** 2021-09-20

**Authors:** Giselle P. Silva, Wania F. Pereira-Manfro, Priscilla R. Costa, Dayane A. Costa, Bianca Ferreira, Daniela M. Barreto, Ana Cristina C. Frota, Cristina B. Hofer, Carlos M. Figueredo, Barbara Coelho, Esper G. Kallas, Lucimar G. Milagres

**Affiliations:** I Departamento de Microbiologia, Imunologia e Parasitologia, Universidade do Estado do Rio de Janeiro, Rio de Janeiro, RJ, BR.; II Divisao de Imunologia Clinica e Alergia, Faculdade de Medicina FMUSP, Universidade de Sao Paulo, Sao Paulo, SP, BR.; III Instituto de Puericultura e Pediatria Martagão Gesteira, Universidade Federal do Rio de Janeiro, Rio de Janeiro, RJ, BR.; IV Departamento de Medicina Preventiva, Faculdade de Medicina, Universidade Federal do Rio de Janeiro, Rio de Janeiro, RJ, BR.; V Departamento de Periodontia, Universidade do Estado do Rio de Janeiro, Rio de Janeiro, RJ, BR.; VI School of Dentistry and Oral Health, Griffith University, QLD, Australia.

**Keywords:** Meningococcal Vaccine, HIV-1 Infection, Bactericidal Antibody, T Cell Exhaustion, CXCL-13

## Abstract

**OBJECTIVES:**

To investigate the expression levels of surface markers of activation (CD38 and HLA-DR), inhibition (PD-1, TIGIT and CD57) and co-stimulation (CD28 and CD127) on CD4^+^ T cells of children/adolescents with vertical HIV infection (HI patients) and HIV-uninfected (HU) controls vaccinated with the meningococcal C conjugate vaccine (MCC).

**METHODS:**

HI patients (n=12), aged 8-17 years, were immunized with two MCC injections, while HU controls (n=9), aged 5.3-10.7 years, received a single MCC dose (as per national recommendation at the time of this study, a single MCC vaccine dose should be given for healthy children and youth aged 1-18 years). The HI patients were categorized according to the combined antiretroviral therapy (cART) treatment. Blood samples were obtained before vaccination, after priming, and after the administration of a booster dose of vaccine to determine the serum bactericidal antibody (SBA) titers and the expression levels of surface markers on CD4^+^ T cells by flow cytometry. The levels of serum cytokines, IL-4 and CXCL-13 were also measured using Luminex kits.

**RESULTS:**

The co-expression of the TIGIT-HLA-DR-CD38 molecules increased in the CD4^+^ T cells of HI patients/no-cART who also showed a lower frequency of CD127^+^CD28^+^ CD4^+^ T cells than HI patients/cART and HU group subjects. There were significant negative correlations between the frequency of exhausted CD4^+^ T cells and the SBA response. IL-4 levels were higher in HI patients/cART and positively correlated with SBA titers but negatively associated with the expression of exhaustion markers. Moreover, the CXCL-13 levels were positively correlated with the exhausted CD4+ T cells.

**CONCLUSION:**

The results of our study suggest that the co-expression of exhaustion markers and/or loss of co-stimulatory molecules influence the SBA response in HI patients.

## INTRODUCTION

The use of meningococcal C conjugate (MCC) vaccine has positively influenced meningococcal disease epidemiology throughout the world (1). In Brazil, case fatality rates of meningococcal disease are as high as 18-24%, and serogroup C meningococcus (MenC) accounted for more than 80% of reported cases before the establishment of routine vaccination (2). In 2006, the Brazilian Ministry of Health recommended that all patients under 13 years of age with human immunodeficiency virus (HIV) infection should be immunized with a single dose of the MCC vaccine. In 2011, Brazil incorporated the MCC vaccine into the routine immunization program, with doses administered at 3 and 5 months of age, with a booster dose at 12 months. In 2014, there was a recommendation for two doses of MCC in children above 12 months of age with HIV infection and adult HIV patients, who were not previously vaccinated (3). Since 2017, an additional dose has been added for all adolescents aged 12-13 years (4). Currently, MenC accounts for approximately 56% of the reported cases in Brazil, 2020 (5). To measure protection against meningococcal disease, the serum bactericidal antibody (SBA) assay is the method of choice (6).

Evidence of the routine immunization of children with HIV-1 infection demonstrates the need for a special vaccine schedule in this population; however, optimal vaccination strategies are yet to be elucidated (7). Understanding the degree of immune recovery after combined antiretroviral therapy (cART) is important in guiding the administration of routine vaccination in patients with HIV-1 (7,8). We have previously shown that the poor antibody responses in cART-treated children and adolescents with HIV infection were associated with a high frequency of CD38+HLA-DR+ CD4+ T cells (9).

During chronic viral infections, high antigenic loads continuously stimulate the T cells, leading to the progressive loss of function, termed as “T cell exhaustion” (10,11). Throughout this period, T cells show an increase in the expression of several inhibitory immune receptors, such as programmed death 1 (PD-1) and T cell immunoglobulin and ITIM domain (TIGIT), which raise the threshold for their activation, resulting in suppressed immune responses (10,11).

Herein, we investigated the frequency of CD4^+^ T cells expressing the inhibitory/stimulatory markers as well as their association with protective antibodies and the expression levels IL-4 and CXCL-13 in the blood of patients with vertical HIV infection (HI patients) and HIV-uninfected controls (HU).

## PATIENTS AND METHODS

### Cohorts

The original study, initiated in 2011, was conducted at Instituto de Puericultura e Pediatria Martagão Gesteira/Universidade Federal do Rio de Janeiro (IPPMG/UFRJ), Rio de Janeiro, Brazil, to investigate the seroconversion rates after MCC vaccination in the HI and HU groups. The inclusion and exclusion criteria used for children enrollment, full information about the cohorts, and ethical approval have been described previously (12). Clinical data were obtained from the medical records of the patients. Blood samples for serogroup C, *N. meningitidis* bactericidal antibodies, and virological and immunological measurements were collected before and after immunization, as described below.

For this study, we selected 12 HI patients and 9 HU children based on the availability of stored peripheral mononuclear blood cells (PBMCs), which were stored in liquid nitrogen for blood collection (2011-2013). Demographic, immunological, and virological parameters for HI participants at baseline were categorized based on cART, while those who were not categorized are shown in Table 1. For the HU cohort, the median (IQR) age was 8.6 (5.3-10.7) years. Eight of the nine HU subjects were men.

### Vaccination and Specimen Collection

After providing informed consent, all patients received one intramuscular injection of MCC (C Polysaccharide/CRM_197_; 10 μg/0.5 mL; Novartis). At the time of the onset of this study, only one dose of the MCC vaccine was recommended in children. However, sue to the low SBA response of the HI patients after only one dose of vaccine, we decided to immunize the children with a second vaccine injection, which was administered approximately one year after the first dose. The HU controls did not receive the booster dose, as per the national recommendation at the time of this study, which specified that only a single MCC vaccine dose should be administered for healthy children and youth aged 1-18 years (13). Blood samples were collected before vaccination (V0), 1-2 months after the first dose (V1), and 1-2 months after the booster dose in the HI group (V2). PBMCs were obtained as described previously (9).

### Serum Bactericidal Assay

SBA assays using a human complement source were performed at the Department of Microbiology, Immunology and Parasitology, Universidade do Estado do Rio de Janeiro, Brazil. Two MenC strains, N753/00 (C:23:P1.22,14-6:F3-9: ST-5122 (cc103)) and N79/96 (C:2b:P1.5-2,10:F5-2: ST-153 (cc8)), representative of two meningococcal disease outbreaks in Brazil, were used as the target strains (9,12). MenC strains were provided by the Adolfo Lutz Institute, Bacteriology Department, Sao Paulo, SP, Brazil.

### Polychromatic Flow Cytometry

**Figure S1:**
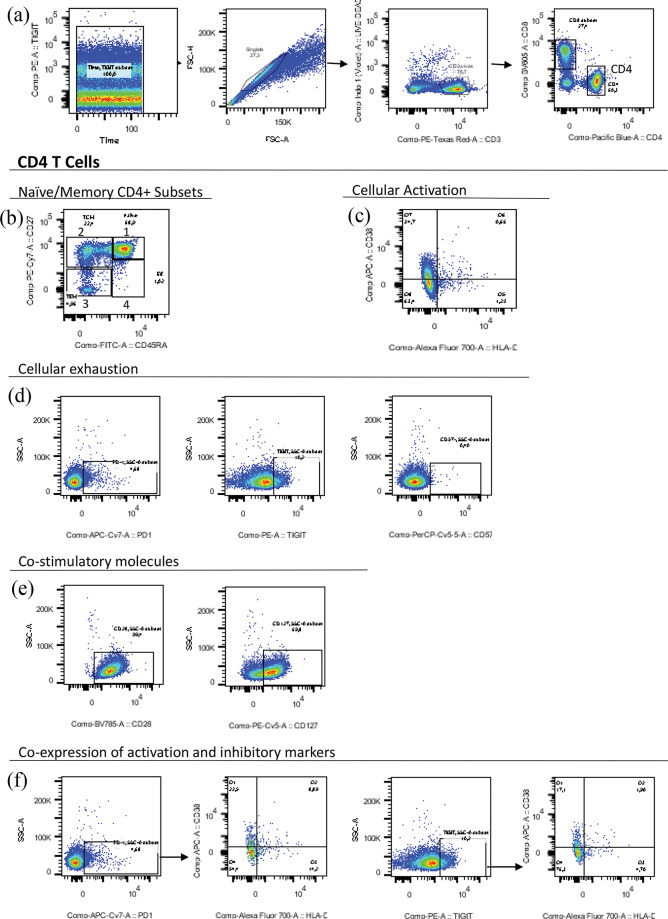
Strategy for the analysis of flow cytometry data from one representative experiment with peripheral mononuclear blood cell (PBMC) samples of responders and non-responders to the MenC vaccine were used to check **(a)** high-quality parameters (Time × PE to check the laser status; Singlets to exclude the doublets cells); Live/Dead x cluster of differentiation (CD)-3-to select living cells positive for CD3 molecule; and the gate CD8 × CD4- to choose CD4^+^ **(b)** Naïve/Memory CD4^+^ Subsets were classified according to the differentiated expression of CD45RA and CD27: 1- Naïve (CD45RA+CD27+); 2- Central Memory (CD45RA-CD27+); 3-Effector Memory (CD45RA-CD27-); 4- Effector cells (CD45RA+CD27-); **(c)** Cellular activation was characterized according to the expression levels of CD38 and human leukocyte antigen (HLA)-DR; **(d)** Cellular exhaustion was characterized according to the expression levels of programmed death 1 (PD-1), T cell immunoglobulin and ITIM domain (TIGIT), and CD57; **(e)** The co-stimulatory molecules investigated were CD28 and CD127; **(f)** Co-expression of activation and inhibitory markers was done first for gating TIGIT or PD-1 CD4+ T cells, and then, gating those positive for DR and CD38. The parameters used for memory T cells were the same as those used for the CD4^+^ T cells (data not shown).

Flow cytometry assays were performed at LIM 60/DCIA/USP, as previously described (9). The monoclonal antibodies used in this study included phycoerythrin (PE)-Texas Red-conjugated CD3 (Invitrogen, Carlsbad, California, USA); Pacific blue-conjugated CD4, PE-Cy7-conjugated CD27, fluorescein isothiocyanate (FITC)-conjugated CD45RA, and Allophycocyanin (APC)-conjugated CD38 (all from BD Biosciences Pharmingen, San Diego, California, USA); PE-Cy5-conjugated CD127, Brilliant violet 785-conjugated CD28, Alexa Fluor 700-conjugated HLA-DR, PerCP-Cy5.5-conjugated CD57, and APC-Cy7-conjugated PD-1 (all from Biolegend, San Diego, California, USA); and PE-conjugated TIGIT (eBioscience, Carlsbad, California, USA). Figure S1 (refer to Appendix) shows the representative dot plots with strategies used to gate the different T-cell populations described in this study.

### Measurement of the Levels of Serum Cytokines

Serum samples collected at baseline (V0) were analyzed to measure the levels of soluble CD14 (sCD14) using standard enzyme-linked immunosorbent assay (ELISA), as described previously (14). Then, the levels of IL-4, IL-10, IL-21, tumor necrosis factor-alpha (TNF-α), interferon (IFN)-γ, and CXCL-13 were measured using commercial Luminex kits (14). The Luminex Platform was from the Department of Periodontics/UERJ.

### Statistical Analysis

Flow cytometry analysis was performed using the FlowJo software v.10 (Tree Star Inc., Ashland, OR, USA). Significance was calculated using the non-parametric Mann-Whitney test. Statistical significance was set at *p*<0.05. The correlation between different measurements described here was analyzed using the non-parametric Spearman rank correlation test. These analyses were performed with the GraphPad-Prism software, version 9 (GraphPad Software, Inc, USA).

## RESULTS

### Baseline Characteristics of the HI Patients

**Figure S2:**
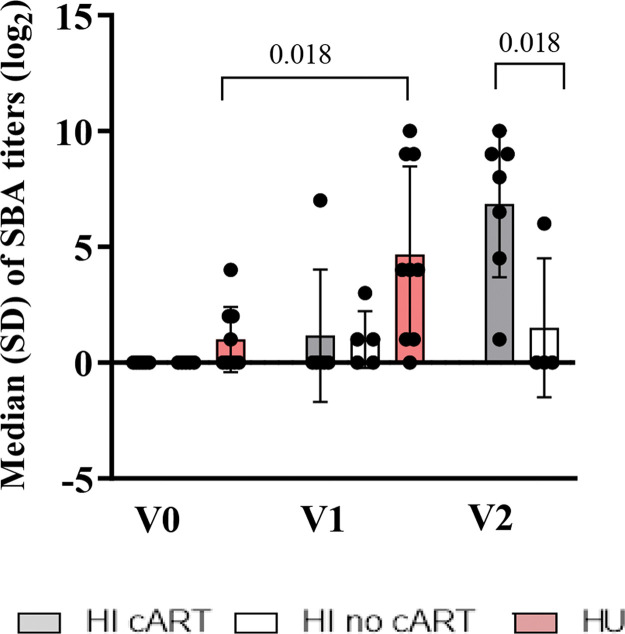
Median titers of bactericidal antibodies against MenC strains in vaccinated HI patients from the cART or no-cART groups and HU children; before (V0), one-two months after the first dose (V1), and after the second dose (V2) of vaccine (MenC-CRM_197_). HIV-uninfected (HU) received only one vaccine injection.

As shown in Table 1, the CD4 count was lower (*p*=0.02) in the HI/no-cART group than in the HI/cART group at baseline. At the time of the onset of this study in 2011, the Brazilian national guidelines for cART initiation for children and adolescents were restricted to patients in the Centers for Disease Control and Prevention (CDC) clinical categories B or C or patients with at least one of the following parameters: CD4 count less than 350 cells/µL, CD4 percentage less than 15%, or viral load>100.000 copies/mL (15). For one patient, cART was initiated approximately six months before the second MCC dose due to a high viral load and decreased CD4 count. SBA titers before vaccination were absent in HI patients, in contrast to the HU cohort (three patients out of nine with SBA titers≥2 (log_2_)). Figure S2 (refer to Appendix) shows the median SBA titers of the three cohorts. HI children needed two vaccine injections to acquire a similar antibody response as detected in the HU cohort after only one vaccination (12,16).

### Increased Co-expression of the TIGIT^-^HLA-DR^-^CD38 Molecules on CD4^+^ T Cells of HI Patients that did not Receive cART

T cell exhaustion is a hallmark of HIV-1 infection (10,11). Here, we investigated the expression of inhibitory molecules (PD-1, TIGIT, and CD57) as well as the expression of activation markers (CD38 and HLA-DR; hereafter described as DR) and co-stimulatory molecules (CD28 and CD127).

**Figure 1:**
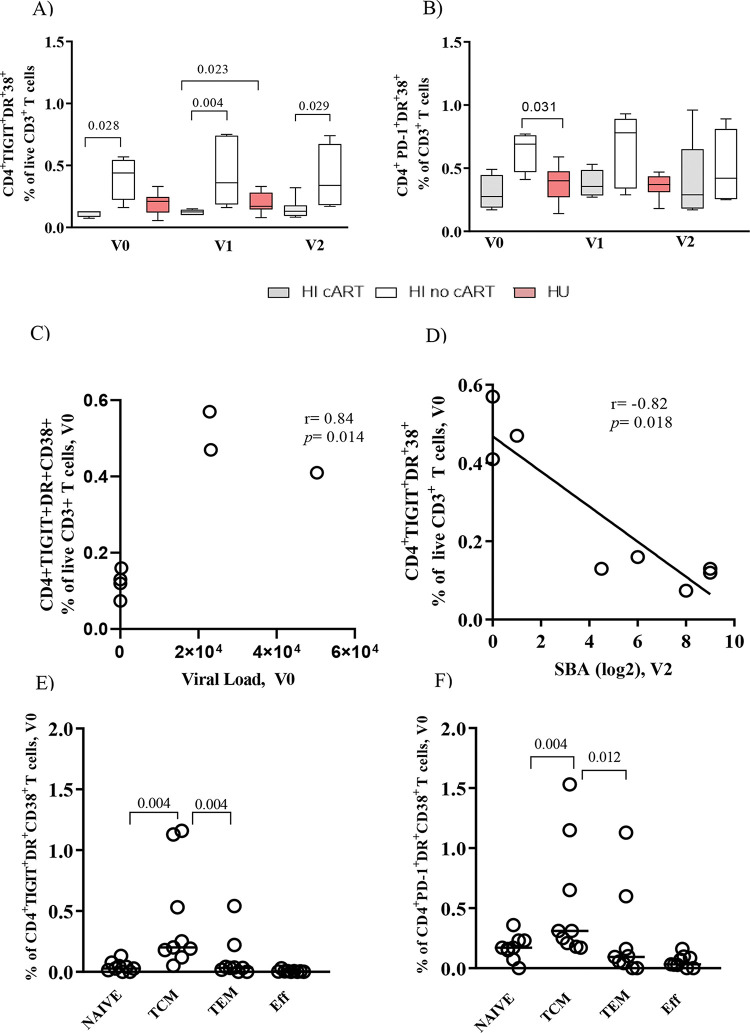
Co-expression of exhaustion (TIGIT or PD-1-DR-CD38) markers on bulk CD4^+^ T cells and TCM in the human immunodeficiency virus (HIV)-infected (HI) or HIV-uninfected (HU) cohorts. Associations with viral load and serum bactericidal antibody (SBA). Frequency of CD4^+^ T cells co-expressing (A) TIGIT-DR-CD38 and (B) PD-1-DR-CD38 at all time points of the study (V0, V1, and V2). Spearman correlation analysis for assessing the correlation between the baseline TIGIT+DR+CD38+CD4+ T cells and (C) Viral load (V0), and (D) SBA titers (V2) for the HI cohort (combined antiretroviral therapy (cART) and no-cART). Frequency of TCM cells co-expressing (E) TIGIT-DR-CD38 and (F) PD-1-DR-CD38 in the HI cohort (cART and no-cART). PBMCs were obtained before vaccination (V0), after one MCC dose (V1), and after two MCC doses (V2). The lines represent the median values. The p-values were calculated using the Mann-Whitney test. Statistical significance was set at *p*<0.05.

Figure 1A shows that the HI/no-cART cohort had a significantly higher frequency of CD4^+^TIGIT^+^DR^+^CD38^+^ T cells than the HI/cART cohort at all time points of the study. A trend toward higher levels of CD4^+^ PD-1^+^ DR^+^CD38^+^ T cells in HI/no-cART is shown in Figure 1B. We found a significant positive correlation (Figure 1C, r=0.84, *p*<0.01) between the frequency of CD4^+^TIGIT^+^DR^+^CD38^+^ T cells and the viral load at baseline, but a negative association (Figure 1D, r=-0.82, *p*=0.02) between the frequency of CD4^+^TIGIT^+^DR^+^CD38^+^ T cells and the SBA after vaccination (V2). The levels of inhibitory and/or activation markers on CD4+ T cells in the HU group were similar to those in the HI/cART group, as shown in Figures 1A and B.

Memory CD4^+^ T cells were classified based on the expression levels of CD45RA and CD27 (Figure S1-b). As shown in Figures 1E and F, TCM was the main subset co-expressing TIGIT-DR-CD38 or PD-1-DR-CD38 in the HI cohort (both the cART and no-cART subjects). The HU cohort showed low levels of CD4^+^ T cells expressing these molecules, without significant differences between the memory subtypes (data not shown). For the HI cohort, significant negative correlations (r=-0.75 to -0.84, *p*=0.03; *p*<0.01) were detected between the frequencies of TIGIT^+^DR^+^CD38^+^ TCM cells measured at all time points studied and the SBA at V2 (data not shown).

### Baseline Serum Levels of IL-4 Positively Correlated with Vaccine-Elicited Protective Antibody Titers and Negatively Correlated with Exhausted CD4^+^ T Cells in HI Patients

**Figure 2:**
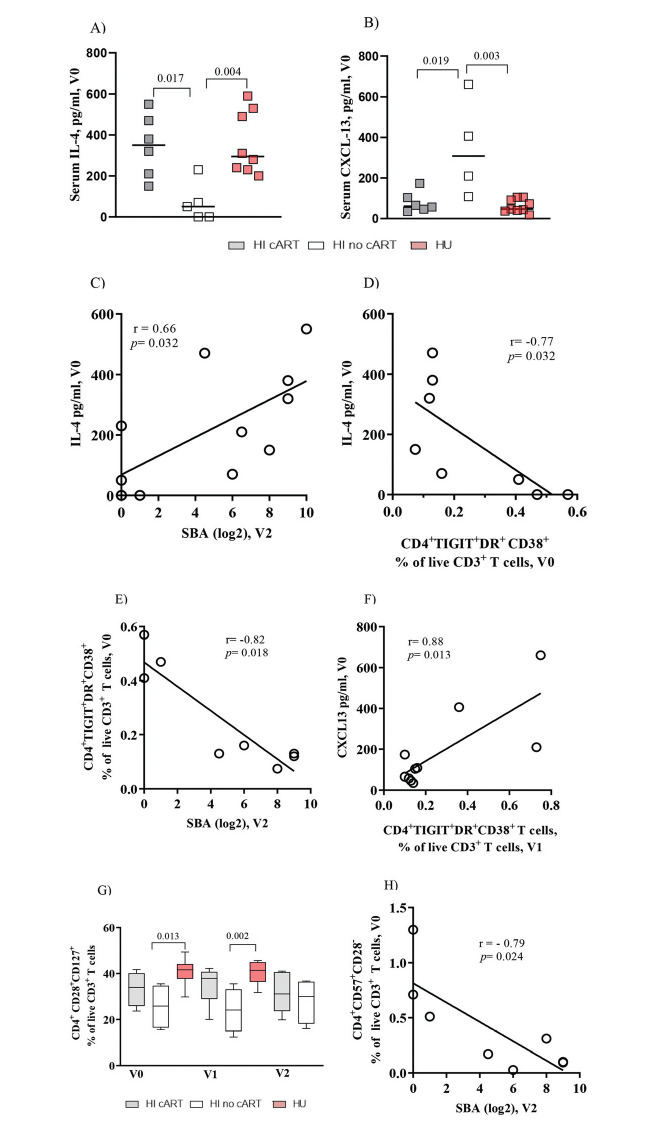
Baseline serum levels of IL-4 are positively correlated with vaccine-elicited bactericidal antibody titers in HI patients. IL-4 and CXCL-13 levels show opposite correlations with the frequency of exhausted CD4^+^ T cells. (A) Pooled data show that the serum IL-4 levels in the HI /cART group (gray closed squares) are significantly higher than those in the HI/no-cART (opened squares) group but are similar to those detected in the HU cohort (red closed squares). (B) The HI/no-cART group (opened squares) shows increased levels of baseline CXCL-13 than the HI/cART group (gray) and HU cohort (red). (C) Baseline blood IL-4 levels of the HI cohort (cART and no-cART) are positively correlated with the SBA at V2 but (D) negatively associated with TIGIT^+^DR^+^CD38^+^CD4+ T cells at V0. (E) In the HI cohort, a negative association between the frequency of CD4^+^ T cells expressing TIGIT/DR/CD38 at V0 with SBA at V2 can be seen simultaneously with (F) a positive correlation of TIGIT^+^DR^+^CD38^+^CD4^+^ T cells at V1 with the CXCL-13 levels at V0. (G) The HI/no-cART group shows a significant reduction of the frequency of CD4^+^ T cells expressing CD28 and CD127 compared with the HU cohort. (H) The lack of CD28 expression together with CD57 expression (V0) negatively associates with SBA at V2 for HI cohort. *p*-values were calculated using the Mann-Whitney test. Spearman non-parametric test was used for correlation analyses. Statistical significance was set at *p*<0.05.

Next, we analyzed the circulating levels of IL-4 and CXCL-13, which are cytokines involved in CD4^+^ T cell-B cell interactions (17). IL-4 levels at entry in the study (V0) were significantly (*p*=0.02 and *p*<0.01) higher in the HI/cART and HU groups, respectively, than in the HI/no-cART group (Figure 2A). In contrast, CXCL-13 levels (Figure 2B) were significantly higher in the HI/no-cART group than in the other two groups.

In the HI patients, we found a significant positive correlation between the baseline serum levels of IL-4 and vaccine-induced SBA titers at V2 (r=0.66, *p*=0.03, Figure 2C). In contrast, a negative association was observed between the IL-4 levels and the frequency of CD4^+^TIGIT^+^DR^+^CD38^+^ T cells (Figure 2D; r=-0.77, *p*=0.03). Consequently, a significant negative association was observed between the frequency of CD4^+^TIGIT^+^DR^+^CD38^+^ T cells at V0 and the SBA titers at V2 (Figure 2E, r=-0.82, *p*=0.02). A negative association was also observed between the proportion of CD4^+^DR^+^CD38^+^ T cells and IL-4 levels (r=-0.820, *p*=0.02; data not shown).

However, the CXCL-13 levels were positively correlated with exhausted CD4^+^ T cells (Figure 2F, r=0.88, *p*=0.01) and with the frequency of CD4^+^DR^+^CD38^+^ (r=0.86, *p*=0.02) or CD4^+^TIGIT^+^PD-1^+^ T cells (r=0.96, *p*=0.01) (data not shown). For the HU cohort, we did not observe any significant correlation between the IL-4 or CXCL-13 levels and the parameters described above. The sCD14 and TNF-α levels were similar for the HI or HU groups, and IFN-γ was detected in the sera of a few individuals, while no IL-10 or IL-21 was detected in any serum sample from our cohorts (data not shown).

These correlation data indirectly suggest that the expression of an exhausted phenotype (TIGIT^+^DR^+^CD38^+^, DR^+^CD38^+^, or TIGIT^+^PD-1^+^) had a negative impact on CD4^+^ T cell function.

### Frequencies of CD4+ T Cells Expressing Co-stimulatory Molecules Are Altered in the HI/no-cART Group

With regard to the co-stimulatory CD28 and CD127 molecules (Figure 2G), the HI/no-cART group showed a significantly lower frequency of CD4^+^CD28^+^CD127^+^ T cells than the HU individuals at baseline (*p*=0.01) and at V1 (*p*<0.01). In humans, senescent T cells typically lose the expression of CD28 and acquire the expression of CD57 (18,19). The expression of CD57 tended to be higher in the HI/no-cART group (data not shown); Figure 2H shows a negative correlation (r=-0.79, *p*=0.02) between CD4^+^CD57^+^CD28^-^ T cell proportion and SBA levels at V2, further indicating the negative impact of the excessive expression of inhibitory molecules on CD4 T cells on the antibody response.

## DISCUSSION

Our study demonstrated that poor specific antibody response to an MCC vaccine and viremia levels were associated with the presence of circulating exhausted bulk or memory (TCM) CD4^+^ T cells (TIGIT^+^DR^+^CD38^+^) in the HI cohort. Patients receiving cART showed a lower frequency of exhausted CD4+ T cells, indicating the importance of early treatment onset. However, the expression of TIGIT and/or PD-1 can persist in infected T cells, even during cART (11,20). TIGIT can indirectly inhibit the T cell responses by inducing tolerogenic dendritic cells and/or acting directly on T cells by attenuating TCR-driven activation signals (21). Taken together, these data suggest that exhaustion of CD4^+^ T cells, particularly, TCM cells, in our HI cohort could be a factor contributing to the need for two vaccine injections to acquire an antibody response comparable to that in the subjects from the HU group (12,16).

The circulating levels of IL-4 and CXCL-13, cytokines involved in the germinal center reaction (17,22) were analyzed. The IL-4 levels were significantly higher in the HI/cART group than in the HI/no-cART group and showed a strong negative association with the frequency of exhausted CD4^+^ T cells, which was positively associated with CXCL-13 levels. These data suggest that low IL-4 and high CXCL-13 levels in the blood of subjects from the HI/no-cART group could be a consequence of a dysfunctional status of exhausted CD4^+^ T cells. Increased CXCL-13 levels may indicate an intense germinal center reaction as a consequence of HIV viremia (20). Although there is no previous evidence, we hypothesized that our findings could be related to the absence of a bactericidal response in about 21% of the HI cohort, even after two doses of the MCC vaccine (16). Unfortunately, we were unable to perform functional assays with isolated lymphocyte subsets to confirm the possible mechanisms contributing to the negative effect of activated/exhausted CD4^+^ T cells on B cell responses. However, our results are in line with published data showing that the B lymphocyte-induced maturation protein-1 (Blimp-1)^+^ T cells exhibiting high expression levels of multiple inhibitory receptors, including PD-1 and TIGIT, are functionally impaired, manifested by low cytokine production and decreased cytotoxicity capacity (23). In a previous report (11), we described a higher activation status (PD-1^hi^CCR7^lo^ICOS^hi^) of CD4^+^ T cells in HI/cART non-responders than in responders to the vaccine. The other limitations of this study include the small number of subjects and that we could not categorize the subjects in our cohorts as responders or non-responders to the vaccine.

Furthermore, herein, we described the fact that the absence of CD28 co-stimulatory molecules together with a positive expression of the inhibitory molecule CD57 in CD4+ T cells from HI patients reflected a negative association with the SBA response to the vaccine. These data suggest a dynamic pattern of the expression of surface inhibitory/activation/stimulatory markers during HIV infection. It has been reported that CD57 expression is associated with proliferation-incompetent CD4^+^ T cells in HI patients (19).

In conclusion, the present study is an observational study indicating that the co-expression of exhaustion markers and/or loss of co-stimulatory molecules influences the bactericidal antibody responses in HI patients. However, further studies are necessary to determine whether the expression levels of TIGIT-DR-CD38 molecules on CD4 T cells and/or blood levels of the cytokines IL-4 and CXCL-13 could be used as potential biomarkers to develop novel vaccine strategies, which in turn, can induce efficient protective antibody responses in HI patients.

## AUTHOR CONTRIBUTIONS

Silva GP, Pereira-Manfro WF, Costa PR and Costa DA performed the flow cytometry experiments. Kallas EG supervised the flow cytometry experiments. Silva GP, Pereira-Manfro WF and Milagres LG performed the bactericidal and ELISA assays. Frota ACC, Ferreira B, Barreto DM and Hofer CB coordinated the recruitment and immunization of patients, collection of biological specimens, and patient follow-up. Milagres LG, Pereira-Manfro WF and Silva GP designed the experiments and analyzed the data. Milagres LG wrote the manuscript. Milagres LG, Silva GP and Pereira-Manfro WF created the Figures and Tables. Frota ACC and Coelho B provided critical review of data and manuscript. Figueredo CM and Coelho B ran the Luminex assays.

## Figures and Tables

**Table 1:** Baseline characteristics of the patients with human immunodeficiency virus (HIV) infection (HI) patients, either receiving or not receiving the combined antiretroviral therapy (cART).

Characteristics	cART (n=6*)	No cART (n=5*)
Age, years Median (Range)	11.4 (8.3-17.2)	15 (9.3-19)
Male (%)	67	40
HIV RNA, copies/mL**, plasma	<50	17492.0 (301-65701)
Nadir CD4%**, blood	13 (1-34)	22 (16-26)
CD4 count**, cells/µL, blood	958 (523-1267)	525 (479-628)
% of CD4**	34 (14-43)	24 (17 to 26)
Length of cART	5.9 (5.4-11.9)	NA
% of CDC clinical category C	83 (5 of 6)	0

NA, not applicable. *For one patient, cART was initiated before the booster dose of vaccine; this patient was moved to the treated group at V2. **median (range).
